# A non-ideal authenticity-based conceptualization of personal autonomy

**DOI:** 10.1007/s11019-018-9879-1

**Published:** 2018-11-19

**Authors:** Jesper Ahlin Marceta

**Affiliations:** Division of Philosophy, KTH Royal Institute of Technology, Stockholm, Sweden

**Keywords:** Autonomy, Authenticity, Anorexia nervosa, Healthcare, Bioethics

## Abstract

Respect for autonomy is a central moral principle in bioethics. The concept of autonomy can be construed in various ways. Under the non-ideal conceptualization proposed by Beauchamp and Childress, everyday choices of generally competent persons are autonomous to the extent that they are intentional and are made with understanding and without controlling influences. It is sometimes suggested that authenticity is important to personal autonomy, so that inauthenticity prevents otherwise autonomous persons from making autonomous decisions. Building from Beauchamp and Childress’s theory, this article develops a non-ideal authenticity-based conceptualization of personal autonomy. Factors that indicate inauthentic decision-making are explicated, and the full concept is defended from three expected objections. The theory is then tested on a paradigm case which has concerned theorists and practitioners for some time, namely the possible inauthenticity of anorexia nervosa patients’ decision-making. It is concluded that the theory seems to be fruitful in analyses of the degree of autonomy of patients’ decision-making, and that it succeeds in providing reliable action-guidance in practical contexts.

## Introduction

Respect for autonomy is a central moral principle in bioethics. The concept of autonomy can be construed in various ways. Under Beauchamp and Childress’s non-ideal conceptualization, everyday choices of generally competent persons are autonomous to the extent that they are intentional and are made with understanding and without controlling influences (2013, p. 104ff). It is sometimes suggested that authenticity, i.e., being “real,” “genuine,” “true to oneself,” or similar, is important to personal autonomy, so that inauthenticity prevents otherwise autonomous persons from making autonomous choices. Yet, while the notion has previously been included in ideal conceptualizations of autonomy, there have at least to my knowledge not been any attempts at incorporating authenticity in a non-ideal conceptualization of personal autonomy.^1^

Elsewhere, I have proposed that judgments of inauthenticity in others are justified under certain conditions (Ahlin 2018b). In this article, I adjust those conditions for the present purposes and add them to Beauchamp and Childress’s account of autonomy. The result is a non-ideal authenticity-based conceptualization of autonomy supplemented with relatively easy detected factors that indicate non-autonomous decision-making.

The article is structured as follows. First, I account for and briefly discuss White’s recently proposed ideal account of authenticity-based personal autonomy. This is followed by a more elaborate explication of Beauchamp and Childress’s non-ideal account. In the subsequent section, I introduce the conditions under which judgments of inauthenticity are justified and add them to Beauchamp and Childress’s account of autonomy to render a non-ideal authenticity-based conceptualization of autonomy. Factors that indicate non-autonomous decision-making are explicated, and three expected objections are met. Then, I apply the complete account to a case which has been thoroughly discussed in the literature on authenticity, namely a patient who suffers from anorexia nervosa and expresses potentially distressing wishes concerning her own medical situation. A brief final section concludes the discussion.

## Personal autonomy

### Ideal accounts of authenticity-based autonomy

There are many different usages of the terms “ideal theory” and “non-ideal theory” (Valentini 2012). Here, I intend “ideal theory” to designate some model—in this case of autonomy and/or authenticity—that is largely hypothetical. Few or no persons or decisions are ever fully autonomous or authentic in this sense, as the conditions under which ideal autonomy or authenticity obtains are perfect or conceptual. By “non-ideal theory,” I intend accounts that are not constructed accordingly. The approach is sometimes also known as “realist” or “problem-oriented,” as it starts from actual people, facts, conditions, etc., in the real world rather than in some theoretical model.

There are various theories aiming to explain authenticity, none of which takes precedence over others (Ahlin 2018a; Noggle 2005). The relevant problem in practical biomedical contexts is not to determine what is authentic, but what is inauthentic. One example of when the notion has been invoked in biomedical contexts is when patients suffering from anorexia nervosa have said that they would rather die than gain weight (Tan et al. 2006). The claim appears to be inauthentic and, arguably, for that reason also non-autonomous (see, e.g., Sjöstrand and Juth 2014). This is discussed at greater length in a below section.

In one of the most recent contributions to authenticity theory in biomedical ethics, White (2018) argues in favor of using the notion of authenticity as a frame of reference in assessments of the validity of patients’ healthcare decisions. More specifically, it should be used in accounts of autonomy as a means to protect high-stake choices from being overridden. White suggests that the notion of authenticity should provide an underlying frame of reference that allows assessments of whether a particular healthcare decision is adequately understood or appreciated. The theory of authenticity which White adopts is broadly Lockean. In it, authenticity concerns the “self,” which should be understood as a set of “enduring, stable overlapping psychological elements, including values, beliefs and desires” (pp. 193–194). This entails that the validity of a patient’s healthcare decisions should be assessed in relation to the historical construction of the patient’s self. A healthcare decision that conflicts with the psychological elements that constitute the patient’s “self” is inauthentic, and this inauthenticity should influence assessments mainly of the patient’s decision-making competence.

Other, less recent, similar ideal theories include Christman (2009) and Juth (2005). Christman argues that “Autonomy involves competence and authenticity; authenticity involves non-alienation upon (historically sensitive, adequate) self-reflection, given one’s diachronic practical identity and one’s position in the world” (2009, p. 155). Juth offers this minimalist definition of personal autonomy: “A person, in a situation, is autonomous to the extent that she does what she decides to do, because she decides to do it, and decides to do what she wants to do, because she wants to do it” (2005, p. 137). He proceeds to argue that, in this analysis, authenticity is one of three components of autonomy (the other two are *decision competence* and *efficiency*) (Ibid).

These suggestions are ideal. White considers some of the practical restrictions in healthcare contexts, such as the epistemic difficulties of determining inauthenticity in others, but builds from a theoretical model rather than from real patients in real contexts. I do not claim that these constructs are mistaken or irrelevant, but I wish to propose a non-ideal alternative to them. In contrast to White’s theory, my proposal is to add the notion of authenticity to a set of conditions which both together and separately indicate that a patient’s decision-making is non-autonomous (in different aspects). This furthers the theoretical approach to autonomy theory which takes authenticity as one of the basic conditions of autonomy, but differs from previous contributions in that it is non-ideal rather than ideal.

### Beauchamp and Childress’s non-ideal account of autonomy

In their book *Principles of Biomedical Ethics* (2013), Beauchamp and Childress propose a non-ideal conceptualization of autonomy. Their account builds on the premise that everyday choices of generally competent persons are autonomous (p. 104). Autonomous actions are then analyzed “in terms of normal choosers who act (1) intentionally, (2) with understanding, and (3) without controlling influences that determine their action” (Ibid). Essentially, the current project is to add a fourth condition to that analysis, namely authenticity. First however, the conditions just mentioned must be further elaborated.

The premise that everyday choices of generally competent persons are autonomous includes standards of incompetence, i.e., conditions that negate a person’s decision-making capabilities. Beauchamp and Childress suggest seven types of related inabilities, including the inability to express or communicate a choice, the inability to understand one’s situation and its consequences, and the inability to understand relevant information (2013, p. 118). These mark a threshold level of decision-making competence, so that persons who display one or more inabilities should be judged as less competent or incompetent to make the decision in question.

The condition of intentional action is explicated through a contrast with accidental action. Acting intentionally requires a plan, i.e., a “representation of the series of events proposed for the execution of [the] action” (p. 104). Accidental actions are not planned accordingly. Intentional actions “correspond to the actor’s conception of the act in question,” whereas accidental actions do not (Ibid).

The condition of understanding means that an act is non-autonomous if the agent does not adequately understand it (Ibid). Having an adequate understanding is different from having a full understanding. For illustration, consider the so-called “butterfly effect,” i.e., a common term designating the fact that even small interventions in a system may have significant effects on an aggregated scale. For instance, flicking a cigarette butt in the dry woods is a small act that may lead to a huge wildfire and thousands of people having to relocate. On Beauchamp and Childress’s account, an agent is not required to have a full understanding of the “butterfly effect” of an act for it to be autonomous. It should not be said that the person flicking the cigarette butt acted non-autonomously because she did not know that the act would have those significant effects. Adequate understanding, i.e., a reasonable estimation of the nature, meaning, and outcome of the act in question, suffices for it to be autonomous.

Instead of spelling out precisely what “adequate” understanding means, Beauchamp and Childress mention factors that may limit understanding, such as “illness, irrationality, and immaturity” (Ibid). When the condition of understanding concerns a person’s informed consent to treatment, Beauchamp and Childress list “the nature and purpose of the intervention, alternatives, risks and benefits, and recommendations” as “typically […] essential” (p. 132). Thus, having an adequate understanding of an act involves awareness of relevant and reasonably foreseeable facts that are central to the act in question. Most importantly, for an act to be autonomous the agent must understand the basics of how it is likely to affect her own person and her way of life.

Finally, the third condition concerns acting without controlling influences (pp. 104–105). Controlling influences may be external to the agent, such as when she is coerced or manipulated into performing some act, or internal to her, such as when she is drunk or suffers from some mental disorder. Obviously, human beings are almost always subjected to some controlling influence. That comes with being a social animal. It is natural to us to lead our lives after the expectation of others, at least to some extent, and our expectations of ourselves are certainly at least partly socially constructed. It is likely that no human being has ever been completely free from controlling influences.

But, Beauchamp and Childress note that controlling influences, unlike the binary notion of intentional and unintentional actions, come in degrees (p. 105). Influences such as coercion and manipulation are controlling to a greater extent than, for instance, the social expectations that women should be beautiful and men should be strong. Because they are more controlling, coercion and manipulation have a greater effect on the autonomy with which an agent acts. Likewise, internal influences such as severe drug addiction may have a greater effect on the autonomy of a person than, for instance, socially contingent self-constraints. Thus, considering only the third and final condition in Beauchamp and Childress’s account of autonomy, an act is autonomous to the extent that it is free from controlling influences.

It should be noted that Beauchamp and Childress adhere to a theory of justification and methodology that builds from John Rawls’s theory of reflective equilibrium (Beauchamp and Childress 2013, pp. 390–429). On my understanding, they hold that a normative claim is justified to the extent that it is coherent with other relevant claims in moral and factual matters, and with our stable and considered intuitions regarding the problem in question. Elsewhere, Beauchamp writes that their method aims to produce “coherent strings of norms that connect basic principles, derivative norms, and context-specific judgments” (Beauchamp and Rauprich 2016, p. 6). In what follows, my arguments should be understood in light of this methodological approach. I return briefly to these methodological comments below in a discussion about so-called “underdetermined” moral concept.

## Authenticity as a condition of autonomous choosing

### Justifying judgments of inauthenticity

In my proposal, judgments of inauthenticity in others concern their decision-making, or more precisely their desires, as desires are the most basic element in ordinary preference-forming and, thus, in decision-making. I follow Taylor (2005), Sjöstrand and Juth (2014), and others in this desire-oriented approach. For reasons of justification, judgments of inauthenticity are delimited to concern only a certain kind of persons, namely those whose medical condition may influence their decision-making so that they hurt themselves or others. Examples of such persons include an anorexia nervosa patient who expresses a wish to die rather than gain weight and someone with a brain tumor that causes him or her to develop pedophilic sexual desires.^2^ For those persons and the desires underlying their healthcare decisions, I argue that it is justified to judge that a desire is inauthentic to the extent that it is due to causal factors that are alien to the person and to the extent that it deviates from the person’s practical identity.^3^

Thus, there are three main elements in my proposal that require some elaboration here; the kind of people and desires included in the analysis, the notion of alien causal factors, and the notion of deviation. For pedagogical reasons, I will go through them in the opposite order. However, first I present some brief notes on the theoretical foundation of my proposal.

In one tradition, the distinguishing feature between authentic and inauthentic desires is whether the desire-holder would endorse her own desires upon critical and informed self-reflection. The tradition is known mainly from Frankfurt (1971) and Dworkin (1988) and has been supported more recently by Juth (2005) and DeGrazia (2005), among others. It has been noted that the distinguishing feature is difficult to observe in others (see, e.g., Ahlin 2018a; Sjöstrand and Juth 2014; and; Swindell 2009). That is, it is difficult to know whether a person would endorse her own desires upon informed and critical self-reflection, and therefore the theoretical ideal is impractical, at best. However, the distinguishing feature can be reversed, so that a desire is inauthentic if the desire-holder would *disapprove* of having it upon critical and informed self-reflection (cf. Juth 2005, p. 153). Then, it is less difficult to observe inauthenticity in others, as empirical factors that indicate that a desire-holder would in fact disapprove accordingly can be identified and articulated in detail. The theory which is presently being spelled out builds on this reversed version of the Frankfurt-Dworkean ideal. The notions of alien causal factors and of deviation are empirical factors indicating that a desire-holder would disapprove of having a desire upon critical and informed self-reflection.

That is the theoretical foundation of my proposal. It is an ideal theory of what distinguishes authenticity from inauthenticity. What follows here, however, does not concern that distinction per se, but the justification of judgments of inauthenticity, i.e., what justifies the judgment that a desire meets the conditions for being inauthentic. That justification is phrased in non-ideal terms, and builds from empirical factors in real persons and contexts.

Consider a person who suddenly displays a desire that is seriously deviating from her practical identity, i.e., the way she usually thinks, behaves, and functions socially (cf. Christman 2009, pp. 149–156). One hypothetical example is Anna, a professional ballet dancer known to love dancing more than anything else, who after being injured refuses to undergo a minor treatment that would enable her to continue dancing (Ahlin 2018a, p. 44). Her refusal builds on desires that are seriously deviating from her practical identity. Therefore, the case invites the thought that Anna’s desires are inauthentic. However, the judgment is not justified. The reasons for why Anna makes the surprising decision to refuse treatment is unknown. Because we do not know the causal history of her desires, we are not justified in making the judgment that they are inauthentic.

Now, consider a 40-year old man who suddenly developed a sexual interest in children that was causally connected to a brain tumor (Burns and Swerdlow 2003). When the tumor was removed the pedophilic symptoms disappeared, and when the symptoms later returned it was found that the brain tumor had grown back. There is no doubt that the tumor caused the man’s sexual interests. Thus, the causal factors of the man’s desires were alien to how he was otherwise construed, which intuitively seems to justify the judgment that they are inauthentic. However, alien causal factors do not suffice to justify that judgment. For instance, sometimes alien empirical factors cause non-alien desires, such as the hypothetical case of a sugar addict who develops a brain tumor causing cravings for sweets. Therefore, it is not justified to make the judgment that an alien desire is inauthentic merely because of its causal history.

The two notions seem to do well when combined, so that desires that are both deviating from the desire-holder’s practical identity and are due to alien causes indicate inauthenticity. However, there is one major flaw in the suggestion that the combination would justify judgments of inauthenticity. Consider a person who displays a deviating and alien desire which is good, in some sense. For instance, in one case, a 90-year old woman who was otherwise quiet and shy suddenly started to make jokes and flirt with young men (Sacks 1985, chap. 11). Her “frisky behavior” was found to be due to untreated syphilis, i.e., an alien cause of deviating desires. But, the woman enjoyed her new self and did not want it to go away. It appears to be unjustified to judge that her desires are inauthentic, in spite of the fact that they are both deviating and due to alien causes.^4^

Therefore, in my proposal, judgments of inauthenticity should be delimited to persons whose medical condition may influence their decision-making so that they hurt themselves or others. Then, it is justified to make the judgment that, for instance, the 40-year old man who developed a sexual interest in children had inauthentic desires while it is not justified to make the judgment that the 90-year old woman who developed a new way of life had inauthentic desires.

Thus, to summarize my proposal:

For persons whose medical condition may influence their decision-making so that they hurt themselves or others, it is justified to judge that an underlying desire is inauthentic to the extent that it is due to causal factors that are alien to the person and to the extent that it deviates from the person’s practical identity.

In the next subsection, I will incorporate it in Beauchamp and Childress’s account of personal autonomy.

### A non-ideal authenticity-based conceptualization of autonomy

The basic premise in the theory is that everyday choices of generally competent persons are autonomous. Call such persons “normal.” A second basic premise is now added: choices made by otherwise normal persons who suffer from some medical condition that may influence their decisions so that they hurt themselves or others are sometimes inauthentic.

Thus, the theory has two basic premises with different functions. The first premise is directly connected to the autonomy of persons; a person who is not generally competent according to the standards of incompetence elaborated on above is less autonomous than a person who is generally competent. However, a person who suffers from some medical condition of the kind discussed here is not necessarily less autonomous than one who does not suffer from such conditions; for instance, the medical condition may not *actually* influence her decisions merely because it can *potentially* do so. Thus, the second premise is only indirectly connected to the autonomy of persons; it enables judgments of inauthenticity through conditions that will be spelled out shortly.

The fourth condition of authenticity may now be added to Beauchamp and Childress’s list, for stylistic reasons between the first and second condition. Thus, autonomous choice can be analyzed in terms of normal persons who act (1) intentionally, (2) from authentic desires, (3) with understanding, and (4) without controlling influences that determine their action. Each condition is assumed to apply until there is reason to believe otherwise. That is, normal persons are assumed to act, e.g., intentionally or with understanding unless something indicates the opposite. It remains here to spell out in practically usable terms factors that indicate that an otherwise normal person acts from inauthentic desires.

Two factors indicate inauthenticity. Both must be present for a judgment of inauthenticity to be justified. In practically useful terms, they read:

**The factor of deviation** It is a factor indicating inauthenticity that the desire under scrutiny does not cohere with how the desire-holder’s identity has developed over time and is presently being sustained.

**The factor of alien causes** It is a factor indicating inauthenticity that the desire under scrutiny is due to causes that are not normal to how the desire-holder is otherwise construed, taking both physical and mental dispositions into consideration.

It should be noted that the factors come in degrees and are sensitive to judgment. For instance, a desire may deviate from a person’s practical identity, but only insignificantly. To illustrate, Anna, the hypothetical professional ballet dancer, may have a desire to drink beer on the evening before an important show. The desire conflicts with her desires to stay focused and do everything that is in her power to perform well on the show, although the deviation from Anna’s desire-set is not significant enough to indicate inauthenticity. That is, Anna may have the authentic desire to drink beer on the evening before an important show. Deviations should be more serious than that to merit the judgment that a desire is inauthentic. Had Anna instead had the desire to try heroin, the judgment may have been different due to the seriousness of the deviation.

Furthermore, as explained above, in this framework judgments of inauthenticity are only justified regarding a certain kind of persons. Therefore, justification of such judgments requires knowledge of the person’s medical condition and substantive deliberation on whether the person may hurt themselves or others. Thus, judgments of inauthenticity are a matter of practical and context-sensitive deliberation in particular cases. Thereby, the present proposal—as Beauchamp and Childress’s original account of autonomy—is conceptually underdetermined. It is a structure for rational deliberation on the authenticity of decisions made by otherwise normal persons, but it does not include complete specifications of how the involved concepts apply in particular cases and contexts. As such, the proposal should be understood not in terms of, e.g., necessary and sufficient conditions, but as generally reason-giving in a framework of reflective equilibrium.^5^

### Objections

In this subsection, I respond to three (internally independent) objections to my proposal; the threshold for making a judgment of inauthenticity appears to be too high, the condition of authenticity brings normative content into an otherwise value-neutral conceptualization of autonomy, and, finally, my suggested addition to Beauchamp and Childress’s concept of autonomy is superfluous.

Because both factors that indicate inauthenticity must be met for a judgment of inauthenticity to be justified, it appears that few actions would ever be judged as inauthentic. This is not a bad thing; the conceptualization of autonomy which is defended here is ultimately anti-paternalist. From an anti-paternalist perspective, it is good that most actions are treated as autonomous and that factors that indicate the opposite are few. What is important is instead that the conceptualization is accurate.

Furthermore, the factors do in fact support judgments of inauthenticity in real cases (see the next section). Consider, for instance, patients suffering from borderline personality disorder (BPD). Some BPD patients are characterized by unstable “selves” and may, for instance, display sudden and dramatic shifts in goals, values, vocational aspirations, types of friends, and so on (Lester 2009). In generic cases, both factors indicating inauthenticity are thus present; BPD patients are otherwise normal persons with seriously deviating desires that are due to alien causes. Their actions and healthcare decisions are non-autonomous, and the present non-ideal conceptualization of autonomy enables the reliable judgment that they are non-autonomous for authenticity-related reasons.

Proceeding with the second objection, it is true that the condition of authenticity brings normative content into the conceptualization of autonomy through the second basic premise, i.e., that choices made by otherwise normal persons who suffer from some medical condition that may influence their decisions so that they hurt themselves or others are sometimes inauthentic. But, the concept was never value-neutral. Most importantly, Beauchamp and Childress’s standards of incompetence are value-laden (2013, pp. 114–20). To paraphrase Buchanan and Brock, the proper standard of incompetence must be chosen; it cannot be discovered (1990, p. 47). Choosing such standards involves moral assessment and deliberation. Thus, any judgement that a person is incompetent to make a certain healthcare decision is moralizing, because the standards of incompetence are morally loaded.

Finally, the third objection is that my suggested addition to Beauchamp and Childress’ concept of autonomy is superfluous; their concept already accounts for concerns of inauthenticity through the condition of controlling influences.^6^ As explained above, Beauchamp and Childress analyze autonomous actions in terms of normal choosers who act “without controlling influences that determine their action” (2013, p. 104). Some controlling influences are internal to the agent, such as, e.g., psychiatric disorders and drug addiction (p. 138). Therefore, the argument goes, as my suggestion builds on the notion of alien causal factors—understood as internally controlling influences—it adds nothing substantial to Beauchamp and Childress’s concept.

However, although they mention the possibility of internally controlling influences, Beauchamp and Childress do not focus on them in their conceptualization of autonomy (pp. 104–105, 138). In fact, internally controlling influences are almost completely left out of the discussion of the condition of non-control. Thus, I see my suggested addition as a contribution to Beauchamp and Childress’s concept as it explicates one kind of internally controlling influence. It enables analysis in one instance of non-control that was previously theoretically underdeveloped. Furthermore, the addition suggests that this kind of internally controlling influence should be understood in terms of authenticity specifically, and not in other terms. Thereby, the addition also connects one kind of internally controlling influences to an already established theoretical school of thought, namely the Frankfurt-Dworkean.

Thus, the three objections that the threshold for making a judgment of inauthenticity is too high, that the condition of authenticity brings normative content into an otherwise value-neutral concept, and that my suggested addition is superfluous do not overthrow my proposed authenticity-based conceptualization of autonomy.

## Testing the theory

### Some methodological remarks

A non-ideal account of autonomy is good only insofar as it provides real normative guidance in practical contexts. Therefore, in this section, I apply the account in an analysis of a healthcare decision made by a person suffering from anorexia nervosa. The person declined medical treatment. The test consists in analyzing whether the desires underlying that decision were inauthentic.

Because of its non-ideal nature, it does not suffice to test the theory on a generic case-description of anorexia nervosa; real testing requires a real case. However, there are no in-depth individual case-descriptions focusing on anorexia nervosa in the bioethical literature on authenticity. Therefore, I have here constructed a hypothetical case building from two interview studies conducted with anorexia nervosa patients, namely Hope et al. (2011) and Tan et al. (2006). The studies have been influential in the bioethical debate on authenticity and are generally considered to be authoritative in this context. The citations below are real but come from different patients in the studies. They are here represented by the hypothetical person “Amy.”

The case-type is chosen because anorexia nervosa is commonly used as a paradigm example of the complexities involved with inauthenticity judgments. The aim when designing the case-token has been to reflect the difficulties of authenticity-related moral problems that are sometimes a reality in healthcare settings. Although the case is purposefully designed for a specific theoretical cause, it is realistic. The realism is central for the present purposes, which is why the case is not designed through mere speculation but is based on empirical studies. To the best of my understanding, “Amy” is a truthful representation of real persons who have been diagnosed with anorexia nervosa.

### The hypothetical Amy

Amy is 25 years old. She has been diagnosed with anorexia nervosa but is now recovered. Two years ago, Amy had a body mass index (BMI) of 17. She then visited a psychiatrist regularly but did not want any physical treatment or medication related to her anorexia. The interview with Amy includes questions which are pertinent to her authenticity and identity, to her decisions and decision-making capacity, and to her values and self-appreciation.

Here, she describes her disorder as separate from her real self (Hope et al. 2011, p. 22):

1) It IS like another voice, it is like another, it’s almost like having two bits of you that are you all the time. The bit of you that is really scared of food and everything that means and the rest of you that wants to be able to get on without it. I just feel like there’s two voices in my head sometimes.

2) So I didn’t really want treatment, but then there’s this little voice deep down inside, which is kind of the complex part, that’s saying “you know you do want treatment really,” but then there’s this kind of overriding big THING which is just like “no, you’re FAT” (laughs), “you don’t need to put on weight!”

Here, Amy describes how her disorder influenced her personality (Ibid, p. 23):

3) I feel like it’s [the anorexia nervosa] made me a meaner person than I was before, … it’s really weird because at some times I can be, like, the most selfless person … and other times I can be completely selfish.

And, here she describes how her desires are conflicting (Ibid, p. 24):

4) But at the moment it’s really hard, I want to eat the normal amounts, but it’s really hard because at the moment, if I did eat the normal amounts I know that I wouldn’t feel happy about it. But I want to be able to.

Here, she describes her anorexia as part of her identity (Ibid, p. 25):

5) Once you’ve taken that [the anorexia nervosa] away, you’ve taken away part of my identity, so I’m bound to feel a bit lost. […] It’s like you’re trying to take away the something that is a huge part of my life, … and if that goes what am I left with?

Here, Amy describes a difficulty to apply a factual belief to her own situation (Tan et al. 2006, p. 271):

6) There’s part of me that didn’t believe it [risk of death], but then I did feel very ill. … Because I didn’t get to an incredibly, incredibly low weight, I wasn’t in hospital, so in which case, I thought, “ok, maybe half a stone down the line that would be very, very true but at the moment I don’t think it’s going to happen.” But also at that point it was a very focused and not very happy life so to be honest I also didn’t care.^7^

This is how Amy answers a question on how important her weight and body size is to her (Ibid, p. 274):

7) I suppose if I were answering the question for anyone else I would probably say it was of no importance, because all my friends are of different sizes and it doesn’t make any difference, but just for me it’s different, I feel like I suppose because I got so caught up in it that it is really important, but I don’t know why, but it is; I feel really guilty of myself, putting weight on it puts on it makes me feel really different.

To summarize, Amy reports of conflicting identities and desires. She explains how the disorder had an influence on her personality, her capability to appreciate the nature of her situation, and on her values and self-appreciation. Now, the analytical task is to make reliable judgments of inauthenticity. When Amy was in this condition she declined medical treatment. Was that decision based on inauthentic desires?

### Analyzing Amy

Amy’s case is complex. She is an adult and under normal circumstances thus both legally and morally entitled to make her own healthcare decisions. Yet, it is clear that there are serious autonomy-related problems involved with her case. It is of interest whether Amy’s healthcare decisions should have been overridden in concern for her well-being. It should be recognized that the outcome of Amy’s case is already known; she is now recovered. This may influence our intuitions. But, the analytical task concerns decisions that Amy made while she was ill, so the outcome must be set aside.

The analysis must begin with answering to what kind of person Amy is and what kind of desires it is that it subject to critical scrutiny. With the theory that is presently being spelled out, it is crucial that Amy’s medical condition was such that it could have influenced her decision-making so that she hurt herself or others, and that her healthcare decision was high-stake accordingly. This was true in Amy’s case, which is reflected in the diagnostic criteria of anorexia nervosa (American Psychiatric Association 2013) and in citations 6 and 7.

To proceed, the substantive analysis of the desires under scrutiny concerns whether they are due to causal factors that are alien to Amy and whether they deviate from her practical identity.

Clearly, anorexia nervosa is one major causal factor. It is a disorder and as such it is alien to how Amy is otherwise construed, taking both physical and mental dispositions into consideration. It is at least partly because of alien causes that Amy declined medical treatment. To some extent, the causal history of Amy’s desires indicate that they are inauthentic, i.e., that she would disapprove of having them upon critical and informed self-reflection.

It is less clear that the underlying desires are incoherent with Amy’s practical identity, that is, how her identity has developed over time and was sustained at the time that she made her healthcare decisions. In citations 1 and 2, she reports of a duality of her personhood, but the descriptions are vague and do not support anything conclusive regarding her practical identity. But, in citation 3 she expresses the view that the disorder had influenced her personality in a way that she did not appreciate. Thus, when she was ill, she held at least some desires that deviated from her practical identity. And, in citation 4 Amy says that there is a “normal” amount of food, here interpreted as normal *to her*, and that it was hard for her to eat so much. This report implies not only that she held desires that were internally conflicting, but also that they conflicted with who she really was. In light of these observations, Amy’s desire to decline medical treatment appear to be deviating and, thus, possibly inauthentic. However, the analysis is not complete yet.

In citation 5, Amy explicitly states that anorexia nervosa is part of her, meaning that the disorder and its influences are not deviating from her practical identity. And, in citation 7, she reports that her weight and body size is very important to her. These values may be due to her anorexia nervosa, but it may also be the case that her anorexia nervosa is due to Amy having these values. In light of these observations, Amy’s desire to decline medical treatment instead appear to be coherent with her practical identity. Or, it is at least not obvious that the desire is incoherent.

Thus, Amy’s desires are partly conflicting with how her identity has developed over time and was sustained at the time that she made her healthcare decisions. To some limited extent, this conflict indicates that the desires are inauthentic, i.e., that Amy would disapprove of having them upon critical and informed self-reflection. However, Amy’s claims that the disorder is part of her and that her weight and body size is very important to her indicate the opposite, i.e., that the underlying desires of Amy’s healthcare decision are in fact authentic. These contradictory indicators should give rise to further investigation and follow-up questions. This is unfortunately impossible in the present case, as there is no more information available. Therefore, although there is some limited evidence supporting the judgment that Amy’s desires are inauthentic, the factors from deviation which are available for analysis are inconclusive.

In conclusion, both factors of alien causes and of deviation are present in the case of Amy. However, because of the epistemic uncertainty involved, which are due to contradictory evidence, it is only justified to some limited extent to judge that the desires underlying her decision to decline medical treatment are inauthentic and, thus, non-autonomous to some degree.^8^

### Evaluating the test

The first thing to be noted is that the analysis is fruitful even though the case is complex, vague, and contains little detailed information. The central authenticity-related moral problems are clearly articulated with solid theoretical support. Both factors that indicate inauthenticity and factors that indicate the opposite are explicated in detail, which enables critical scrutiny. Also, the results appear to be generally reason-giving in a framework of reflective equilibrium. That is, to some limited extent, the analysis supports the judgment that Amy’s decision was non-autonomous for authenticity-related reasons. The theory is successful in these respects, mainly because it provides a conceptual framework that enables detailed analysis.

Furthermore, it seems reasonable to assume that the theory could be even more fruitful in future analyses of similar cases, as it provides theoretical support for focusing on a certain kind of behavior in patients and for asking them certain kinds of questions. It provides a reliable framework for analyses of personal autonomy in terms of authenticity.

## Concluding remarks

In this article, the Frankfurt-Dworkean tradition of thinking about authenticity has been merged with Beauchamp and Childress’s non-ideal account of autonomy. The result is a non-ideal authenticity-based account of autonomy that, when applied, seems to be fruitful in analyses of the degree of autonomy of patients’ decision-making in healthcare. Thereby, the theory succeeds in providing reliable and practical action-guidance in a matter which has concerned theorists and practitioners for some time.

Because it provides reliable action-guidance, the theory may be foundational for paternalist considerations of coercive care. However, such considerations must include normative support concerning the sufficient degree of epistemic certainty of inauthenticity and the justified proportionality of the intervention, among other things. Thus, although the present theory may be foundational for paternalist interventions in the name of authenticity, it does not by itself provide sufficient moral support for coercive care.

## Footnotes

1 One possible exception is Swanson, who argues that a non-ideal conceptualization of autonomy should include “a consistent sense of personal identity” (2017, pp. 51–53).

2 The example with the pedophilic desires is not hypothetical; this happened to an otherwise normally functioning adult man. See Burns and Swerdlow (2003) and the below.

3 This proposal builds on Ahlin (2018b) but includes other kinds of persons and desires. In Ahlin (2018b), persons that are known to wish to adhere to the prevailing social and moral standards and desires that are seriously undesirable according to those standards are justifiably targeted by judgments of inauthenticity. I will not elaborate further on this difference here.

4 It may also be noted that desires can be inauthentic although the available empirical evidence does not suffice for observers to be justified in making that judgment.

5 For a more in-depth discussion of how underdetermined moral concepts should be applied in practical contexts, see Beauchamp and Rauprich (2016).

6 I am grateful to an anonymous reviewer of an earlier version of this article for pointing this out.

7 1 stone = 14 lb = 6.4 kg.

8 Citation 6 reflects that Amy’s decision may have been non-autonomous to some extent also in the sense that she had limited decision-making capabilities, but it is beyond the present purposes to elaborate on this observation.
